# Activity of two key toxin groups in Australian elapid venoms show a strong correlation to phylogeny but not to diet

**DOI:** 10.1186/s12862-020-1578-x

**Published:** 2020-01-13

**Authors:** Theo Tasoulis, Michael S. Y. Lee, Manon Ziajko, Nathan Dunstan, Joanna Sumner, Geoffrey K. Isbister

**Affiliations:** 1 Clinical Toxicology Research Group, University of Newcastle, Newcastle, New South Wales 2308 Australia; 2 Earth Sciences Section, South Australian Museum, North Terrace, Adelaide, S.A 5000 Australia; 3 College of Science and Engineering, Flinders University, Bedford Park, S.A 5042 Australia; 4 Venom Supplies, Tanunda, South Australia 5352 Australia; 5 Museums Victoria, Carlton Gardens, Carlton, VIC 5053 Australia

**Keywords:** Snake, Evolution, Phylogeny, Venom, Snake venom, Toxin, Diet, Phospholipase

## Abstract

**Background:**

The relative influence of diet and phylogeny on snake venom activity is a poorly understood aspect of snake venom evolution. We measured the activity of two enzyme toxin groups – phospholipase A_2_ (PLA_2_), and L-amino acid oxidase (LAAO) – in the venom of 39 species of Australian elapids (40% of terrestrial species diversity) and used linear parsimony and BayesTraits to investigate any correlation between enzyme activity and phylogeny or diet.

**Results:**

PLA_2_ activity ranged from 0 to 481 nmol/min/mg of venom, and LAAO activity ranged from 0 to 351 nmol/min/mg. Phylogenetic comparative methods, implemented in BayesTraits showed that enzyme activity was strongly correlated with phylogeny, more so for LAAO activity. For example, LAAO activity was absent in both the *Vermicella* and *Pseudonaja/Oxyuranus* clade, supporting previously proposed relationships among these disparate taxa. There was no association between broad dietary categories and either enzyme activity. There was strong evidence for faster initial rates of change over evolutionary time for LAAO (delta parameter mean 0.2), but no such pattern in PLA_2_ (delta parameter mean 0.64). There were some exceptions to the phylogenetic patterns of enzyme activity: different PLA_2_ activity in the ecologically similar sister-species *Denisonia devisi* and *D. maculata*; large inter-specific differences in PLA_2_ activity in *Hoplocephalus* and *Austrelaps*.

**Conclusions:**

We have shown that phylogeny is a stronger influence on venom enzyme activity than diet for two of the four major enzyme families present in snake venoms. PLA_2_ and LAAO activities had contrasting evolutionary dynamics with the higher delta value for PLA_2_ Some species/individuals lacked activity in one protein family suggesting that the loss of single protein family may not incur a significant fitness cost.

## Introduction

**Fig. 1:**
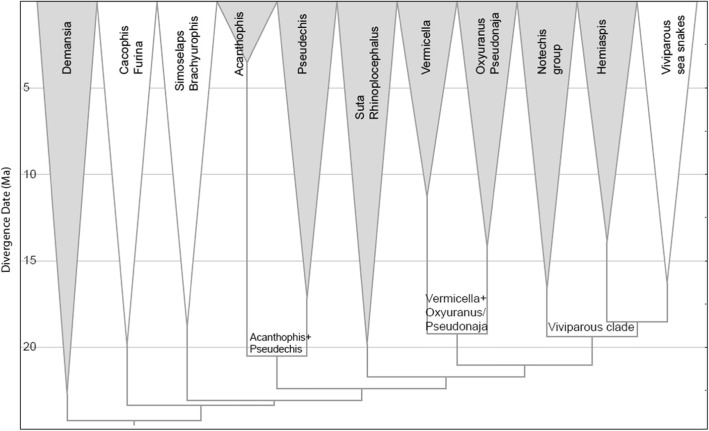
Phylogeny of Australian elapids showing the 11 major clades. The eight shaded clades were sampled in this study

Venomous snakes in Australia belong almost entirely to the front-fanged family Elapidae. The diversity of this family is the result of a continental scale adaptive radiation thought to be approximately 25 million years old [1]. This entire Australasian radiation (including New Guinea and the Solomons), currently consists of approximately 120 terrestrial and more than 60 marine species of snakes [2]. A recent phylogeny divided the Australasian elapid radiation into 11 major clades [1]. *Demansia, Furina/Cacophis, Simoselaps/Brachyurophis, Acanthophis, Pseudechis, Rhinoplocephalus/Suta, Vermicella, Pseudonaja/Oxyuranus, Notechis, Hemiaspis* and *Hydrophiini* (viviparous sea snakes) (Fig. 1).

Snake venoms are mixtures of different protein families. A recent review of snake venom proteomes globally [3], identified four major and six secondary protein families that account for the majority of proteins in both elapid and viperid venoms. Australasian elapid venoms contain all four major protein families; three-finger toxins (3FTx), phospholipase A2 (PLA2), snake venom serine protease (SVSP), and snake venom metalloprotease (SVMP) – and all six secondary protein families; disintegrin (DIS), L-amino acid oxidase (LAAO), natriuretic peptides (NP), kunitz peptides (KUN) cysteine-rich secretary proteins (CRiSP) and C-type lectins (CTL) [3]. These protein families are typically unevenly represented in the venom proteome of a particular species. We identified two protein families of interest, the major protein family PLA_2_, and the secondary protein family LAAO. These particular protein families were chosen as of the 10 major protein families present in snake venoms, only four are enzymatic. Two of these (SVMP and SVSP) were not included in the study. SVMP is not a major component of Australasian snake venoms, while SVSP is a protein family with multiple sites of enzyme activity which would require multiple different types of assays. PLA2s can be mono or multimeric with a molecular mass of 12 to 18 kDa per monomer [4–7]. They have a highly diverse toxicological profile – including pre-synaptic neurotoxicity [5, 7], myotoxicity [8] and anticoagulant activity [9]. PLA2 enzymes catalyze the hydrolysis of the ester bond at the *sn*-2 positon of glycerophospholipids releasing lysophospholipids and fatty acids [4]. The toxic effects of LAAO proteins are imperfectly understood as the literature contains conflicting reports [10, 11]. However, its three-dimensional structure and biochemistry have been elucidated. LAAO is a homodimer with the molecular mass of each monomer being 50 to 70 kDa [12]. LAAO oxidizes an amino acid substrate to form an imino acid which then undergoes non-enzymatic hydrolysis yielding α-keto acid and ammonia. The first step of this process causes a reduction of flavin adenine dinucleotide (FAD) which is then oxidized in the presence of molecular oxygen to form H_2_O_2_ [12].

Studies on snake venom evolution have largely focussed on comparing the sequence similarity of amino acids in homologous toxins between related snake species (eg. 3FTxs in the colubrid *Boiga* [13], and Australian elapids [14, 15], PLA_2_s in the viperids - *Vipera* [16], *Gloydius* [17] and *Crotalus* [7]), and tracing the molecular evolution of toxins among and between snake genera (eg. SVMPs [18, 19] and CRiSPs [20]). Far less is understood about the temporal dynamics of snake venom evolution, whether venom composition is more strongly influenced by phylogeny or diet, and the importance of genetic drift in snake venom evolution. By combining published studies on venom proteomes with published phylogenies it can be shown that the venoms of some snake genera co-vary with phylogenetic distance. An example of this is the venom proteomes of the viper genus *Bitis* [21], which closely mirror the phylogeny of this genus as proposed by Wittenberg et.al [22]. .Several studies have demonstrated prey-specific toxicity in snake venom [23, 24]. An example of this is the prey specific venom of the colubrid snake *Boiga irregularis* which has been shown to be highly toxic to birds and lizards - its primary prey [25], while being far less toxic to mammals [26]. Conversely one study on an Australian elapid (*Notechis scutatus*), showed no correlation between venom and diet [27], and suggested that genetic drift was responsible for observed variation in venom profiles [28].

The Australasian elapid radiation presents an excellent opportunity to investigate the complexities and temporal dynamics of snake venom evolution as it is extremely speciose, and contains species with highly divergent morphological and dietary specializations. This radiation includes species that have adapted to an extreme range of habitats, from deserts to rainforests and tropical grasslands to subalpine woodlands. The diet of Australian elapids is well understood as a result of a series of studies by Shine (e.g. [29, 30], see Additional file 1: Table S1. Supplementary section). Lizards are a major part of the diet of most snake genera/species, particularly the lizard family Scincidae [29, 31–34] . There have been several adaptive moves away from a dietary reliance on lizards,- resulting in snake species that specialize in preying on frogs [30, 35], mammals [36], squamate eggs [37] and other snakes [38]. In addition many Australasian elapid genera include dietary generalists [39–41].

We aimed to investigate the evolution of two protein families, PLA_2_ and LAAO, in Australasian elapid venoms, by measuring their enzymatic activity, and analysing this with respect to phylogeny and snake diet. The study included 90 individual snakes from 17 genera, with representatives from eight of the 11 major clades. We performed ancestral state reconstructions and evaluated their evolutionary dynamics using phylogenetic comparative methods, and compared venom activity at several taxonomic hierarchies - major clades, inter-generic and intra-generic.

## Results

### Phospholipase A_2_

**Fig. 2:**
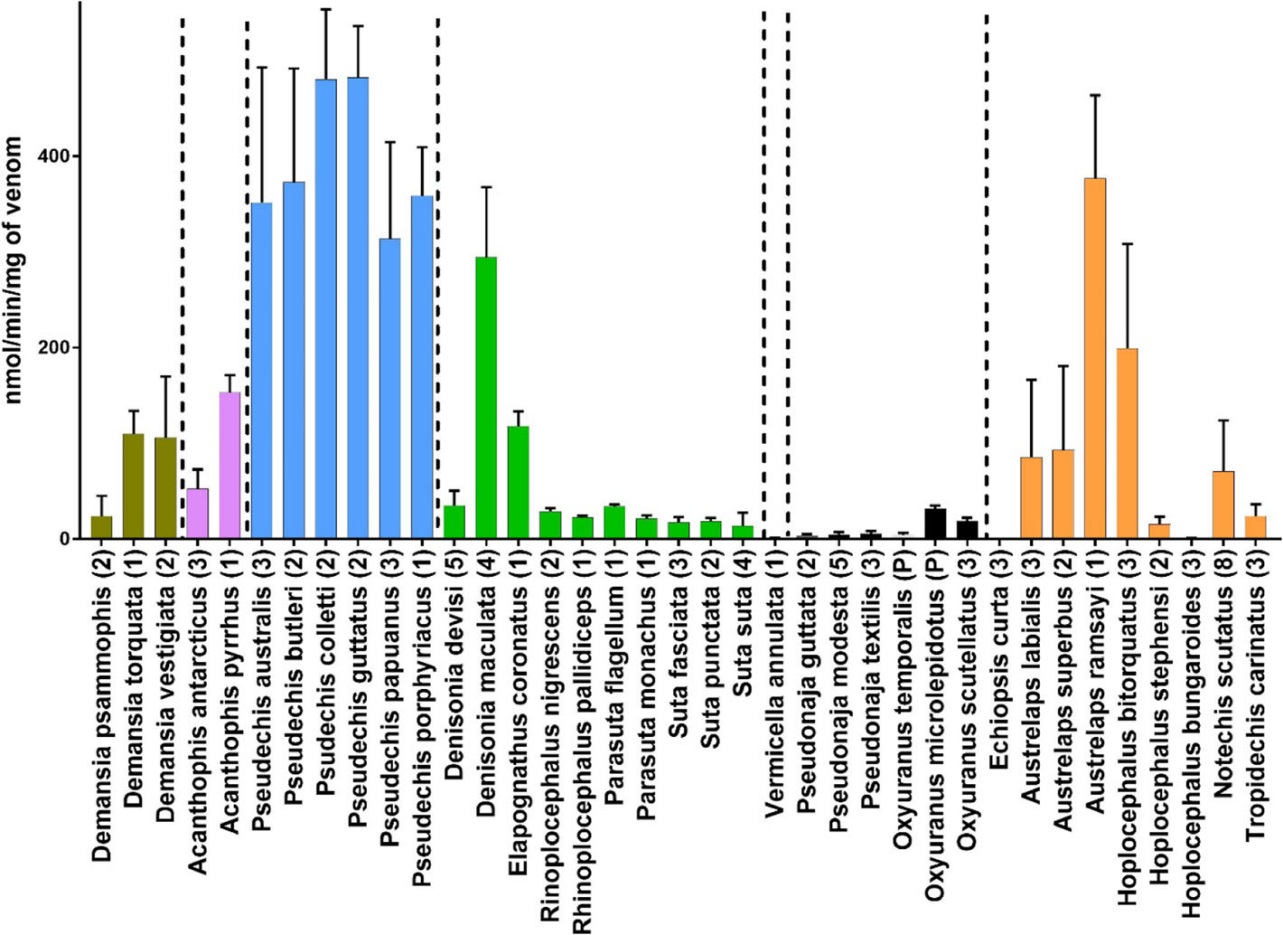
A bar chart (mean with SD for technical replicates), showing PLA_2_ activity for 37 species of Australian elapids representing seven major clades. Vertical dashed lines and bar colours separate major clades as determined by Lee *et.al* 2016. Y-axis units are nanomoles of chromophore released per minute per milligram of venom. All assays were replicated *n* = 5. Numbers in brackets indicate numbers of individuals assayed and (P) indicates pooled venom

PLA_2_ activity was measured in 90 individual snakes from 37 different species (Fig. 2). PLA2 activity values ranged from 0 to 526 nanomoles of chromophore produced per minute per mg of venom (nmol/min/mg; averaged values for individual snakes). Activity levels were arbitrarily defined as 0 to 25 nmol/min/mg = low, 25 to 100 nmol/min/mg = medium, 100 to 300 nmol/min/mg = high and > 300 nmol/min/mg = very high. PLA2 activity was present in all species tested, except the monotypic genus *Echiopsis*. The *Demansia* clade had high PLA_2_ activity, with the highest activity for the genus being *D. torquata* (110 nmol/min/mg). The *Acanthophis* clade had medium to high PLA_2_ activity. It was high in *Acanthophis pyrrhus* (153 nmol/min/mg), and medium in *A. antarcticus* (53 nmol/min/mg)*.* The *Pseudechis* clade had the highest PLA_2_ activity of any clade, with the sister species *Pseudechis.colletti*/*P.guttatus* having the strongest PLA_2_ activity of any species – *P.colletti* (480 nmol/min/mg) and *P. guttatus* (481 nmol/min/mg). The *Rhinoplocephalus*/*Suta* clade had low to medium PLA_2_ activity, except for high levels in *Denisonia maculata* venom (294 nmol/min/mg) and *Elapognathus coronatus* venom (118; nmol/min/mg).Only one species, *Vermicella annulata* was tested in the *Vermicella* clade which had almost no activity (1 nmol/min/mg). The *Pseudonaja*/*Oxyuranus* clade had low PLA_2_ activity, although *Oxyuranus* had higher activity than *Pseudonaja*. The *Notechis* clade had very high variability in PLA_2_ activity, ranging from undetectable in *Echiopsis,* to very high for *Austrelaps ramsayi* (376 nmol/min/mg). The three species in the genus *Hoplocephalus* showed a strong non-overlapping range of inter-specific variability in PLA_2_ activity - high in *H. bitorquatus* (199 nmol/min/mg), low in *H. stephensi* (16 nmol/min/mg), and barely detectable in *H. bungaroides* (0.73 nmol/min/mg).

### L-amino acid oxidase

**Fig. 3:**
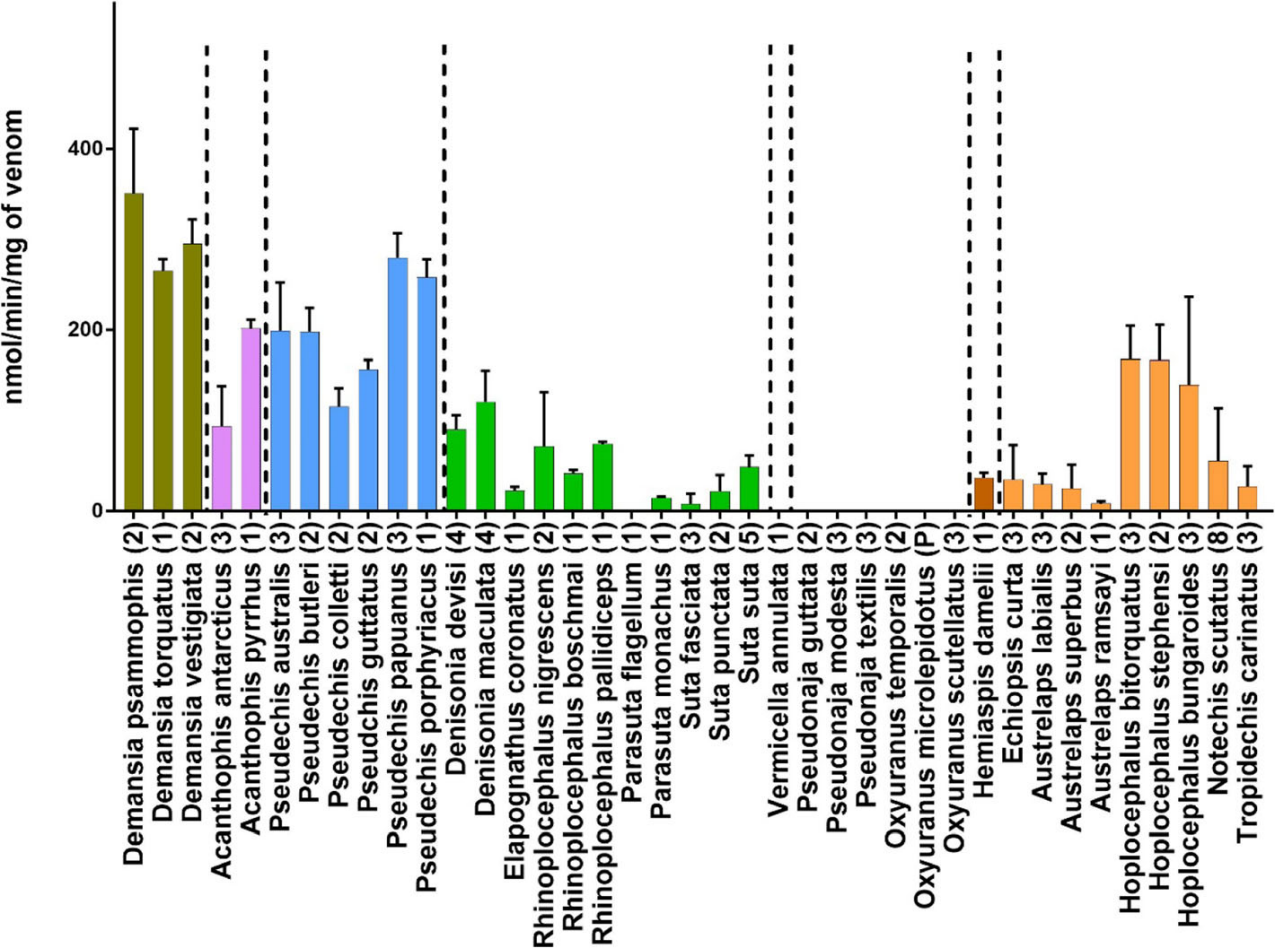
A bar chart (mean with SD), showing LAAO activity for 39 species of Australian elapids representing eight major clades. Vertical dashed lines separate clades as determined by Lee *et. al.* 2016. Y-axis units are nanomoles of H_2_O_2_ released per minute per milligram of venom. All assays were replicated *n* = 5. Numbers in brackets indicate numbers of individuals assayed and (P) indicates pooled venom

LAAO activity was measured in 90 individual snakes from 39 species (Fig. 3). LAAO activity values ranged from 0 to 410 nanomoles of H_2_O_2_ produced per minute per milligram of venom (nmol/min/mg; averaged values for individual snakes). Activity levels were arbitrarily defined as 0 to 50 nmol/min/mg = low, 50 to 100 nmol/min/mg = medium, 100 to 250 nmol/min/mg = high and > 250 nmol/min/mg = very high. The *Demansia* clade had the highest activity, particularly *Demansia psammophis* (351 nmol/min/mg). Activity in the *Acanthophis* clade was medium in *A. antarcticus* (94 nmol/min/mg), but high in *A. pyrrhus* (201 nmol/min/mg). The *Pseudechis* clade also had high LAAO activity, with *P.papuanus* having the highest activity in the genus (279 nmol/min/mg). The *Rhinoplocephalus*/*Suta* clade had low to medium activity (typically about 50 nmol/min/mg), with the species *D. maculata* again having the highest activity in the clade (121 nmol/min/mg). Some individuals in this clade entirely lacked LAAO activity (*Parasuta flagellum* and *Suta fasciata*). *Vermicella* and *Pseudonaja*/*Oxyuranus* clades had no LAAO activity. LAAO activity in the *Notechis* clade was low for all genera (typically in the range of 25 to 30 nmol/min/mg), with the notable exception of the genus *Hoplocephalus* which showed relatively high activity for all species*, H. bungaroides* (139 nmol/min/mg), *H.stephensi* (167 nmol/min/mg) and *H. bitorquatus* (168 nmol/min/mg).

### Phylogenetic relationships of PLA_2_ and LAAO enzyme activity

**Fig. 4:**
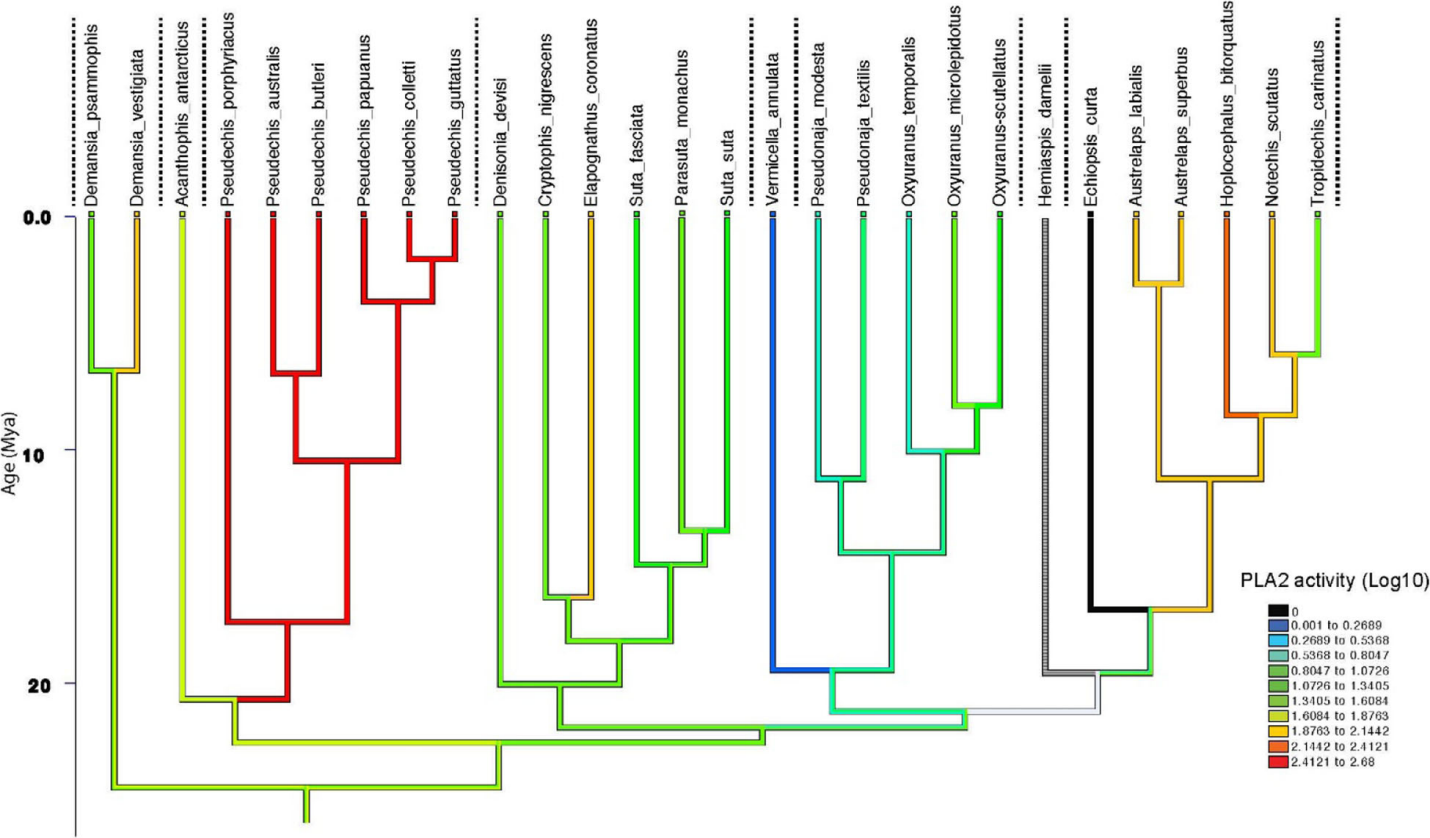
PLA2 activity reconstructed using linear parsimony on a phylogeny [1] of 16 of the 17 genera tested (27 of the 37 species; *Hemiaspis* has no PLA2 data). The Y-axis represents millions of years before present. Values had 1 added before log transformation (to avoid attempting to log 0 values, as any species with activity below the threshold of detection of 0.5 –unlogged value -was allocated a score of 0). Dotted vertical lines separate clades. Warmer colours (red) show higher activity, black is no activity. White line on lower left of the graph is due to *Hemaispis* having no data

**Fig. 5:**
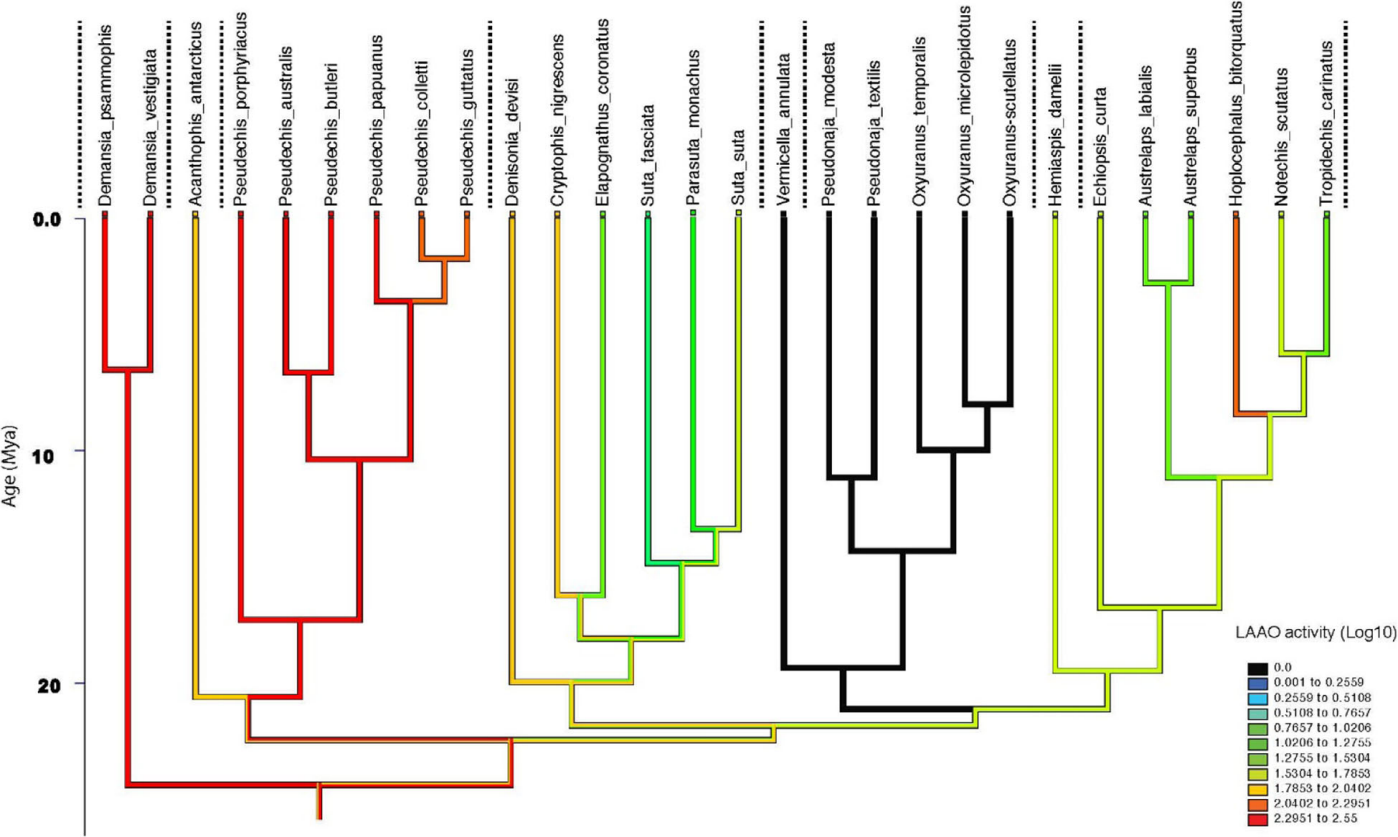
LAAO activity reconstructed using linear parsimony on a phylogeny [1] for all 17 genera tested (28 of the 39 species for which activity was measured). Y-axis represents millions of years before present. Values had 1 added before log transformation (to avoid attempting to log 0 values, as any species with activity below the threshold of detection of 0.5 –unlogged value - was allocated a score of 0). Dotted vertical lines separate clades. Warmer colours (red) show higher activity, black is no activity

Linear parsimony showed that there was a strong association between both PLA_2_ activity and LAAO activity and the currently accepted phylogenetics of the eight major clades of elapids tested (Figs. 4 and 5).

Ancestral state reconstruction of PLA_2_ estimates medium PLA_2_ activity levels (29–53 nmol/min/mg, i.e. 1.6 to 1.8 log transformed), at the commencement of the Australian elapid radiation. There was then an accelerated early burst of evolution at the base of the *Pseudechis* clade, which has been retained in all species of this clade (Fig. 4). There was also an early burst of accelerated evolution for PLA_2_ at the base of the *Notechis* clade, which occurred after the divergence of *Echiopsis* (in which PLA_2_ activity has been lost). There was an early almost total loss of activity in the *Vermicella* clade. Overall, the remaining clades were stable, without changes in PLA_2_ activity, except for isolated taxa. There were isolated increases in PLA_2_ activity for *Demansia vesigiata* (*Demansia* clade), *Elapognathus coronatus* (*Rhinoplocephalus/Suta* clade), and *Hoplocephalus bitorquatus* (*Notechis* clade). Additionally there were two other instances of increased PLA_2_ activity not incorporated into the tree due to lack of molecular data – *Denisonia maculata* and *Austrelaps ramsayi* (Fig. 2).

Ancestral state reconstruction for LAAO activity estimates high activity levels of 94–371 nmol/min/mg, i.e. 1.98–2.55 log transformed at the commencement of the Australian elapid radiation). These high activity levels remained stable in the early diverging clades *Demansia* and *Pseudechis* (Fig. 5). There was a loss of activity in the remaining clades, with a total loss of activity in the *Vermicella* and *Pseudonaja/Oxyuranus* clades, which appears to predate the divergence of these sister clades. There has been a variable reduction of LAAO activity in several taxa in the *Rhinoplocephalus*/*Suta* clade and in the *Notechis* clade. There has been a late burst of accelerated evolution in the genus *Hoplocephalus* which occurred before the subsequent speciation within this genus. This increase in activity was unique among genera in the *Notechis* clade.

**Fig. 6:**
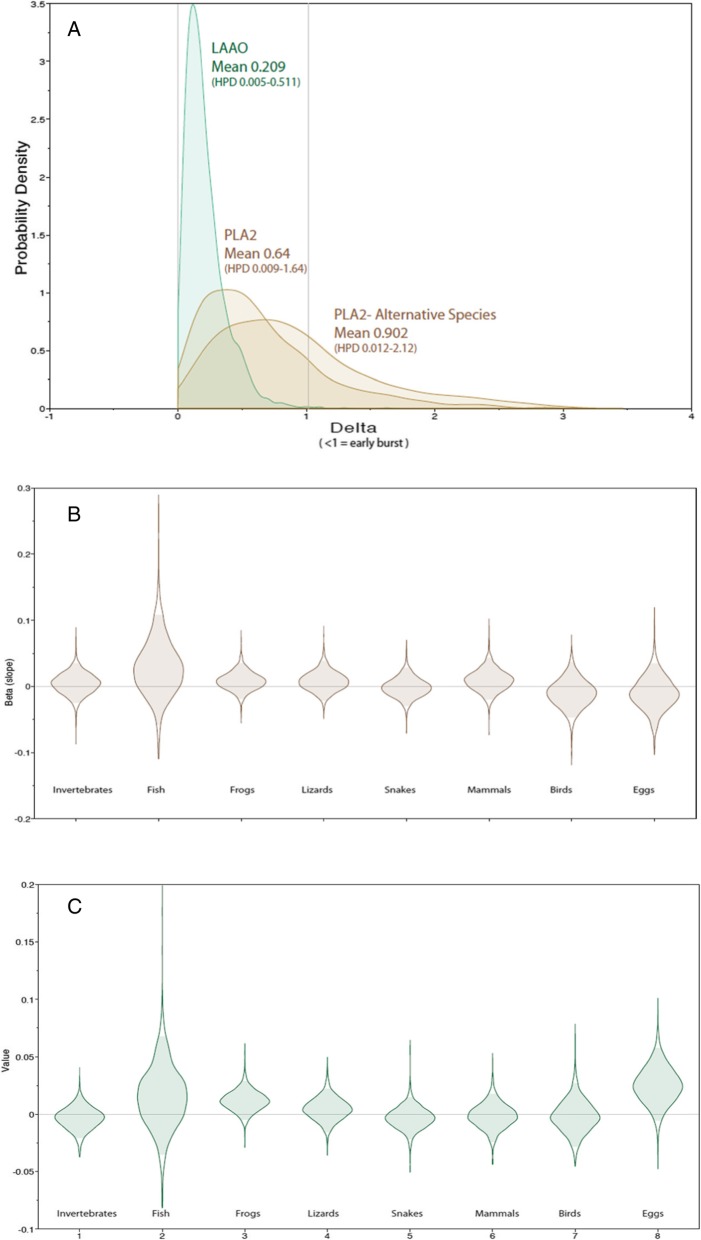
Testing for accelerated evolution of PLA_2_ and LAAO, and for correlation with diet, based on phylogenetic comparative methods in BayesTraits. (Upper) The delta parameter estimates for PLA_2_ and LAAO: values <1 indicate faster initial rates of change. We have included an adjusted PLA_2_ profile (alternative species) with venom values for *Denisonia maculata* and *Austrelaps ramsayi* substituted for their sister-species, which changes our delta value from 0.64 to 0.9. (Middle and Lower) Regression coefficients (beta) values for PLA_2_ and LAAO when phylogenetically regressed against the proportions of eight dietary items. A beta of 0 indicates no correlation

The linear parsimony optimizations (using species means) are shown in Fig. 6; square-change parsimony (which is very similar to maximum likelihood [42]), yielded similar patterns (see Additional file 1: Figures S1 and S2). There was no evidence for accelerated rates of evolution in PLA_2_ with an estimated Delta parameter of 0.64 (95% Highest Posterior Density interval [=HPD] 0.009–1.64) but strong evidence for this in LAAO with a value of 0.209 (95% HPD 0.005–0.511) (Fig. 6). Values of less than 1 indicate faster early rates of evolution.

### Dietary categories and enzymatic activity

Activity levels of PLA2 and LAAO showed no evidence of being correlated with the eight dietary categories; invertebrates, fish, frogs, lizards, snakes, mammals, birds and eggs. In nearly every comparison, the regression coefficient (beta) was close to 0 and the 95% HPD always included 0 (Fig. 6BC). The only potential associations were a weak (i.e. non-significant) positive correlation between LAAO activity and the amounts of frogs and eggs in the diet.

## Discussion

We have demonstrated a strong phylogenetic signal in PLA_2_ and LAAO activity in the Australian elapid radiation by measuring these enzymes in eight of the 11 currently accepted major clades. We found no relationship between PLA_2_ and LAAO activity and dietary preferences within this phylogenetic framework. We have also demonstrated that these two toxin families exhibit contrasting evolutionary dynamics, with LAAO characterized by early burst accelerated evolution and PLA_2_ showing consistent rates of evolutionary change throughout the timespan of the Australasian elapid radiation. Activity for both protein families was remarkably clade specific.

### PLA_2_ activity

Our ancestral state reconstruction suggests that evolutionary rates of PLA_2_ activity has remained relatively constant throughout the Australian elapid radiation. Early major shifts occurred independently in the *Pseudechis* and *Notechis* clades and *Elapognathus coronatus.* We recorded four instances of late shifts in PLA_2_ activity; *Demansia vestigiata* (*Demansia* clade), *Denisonia maculata* (*Rhinoplocephalus*/*Suta* clade), and *Austrelaps ramsayi* and *Hoplocephalus bitorquatus* (*Notechis* clade). This suggests that PLA_2_ is a dynamic protein family still under positive selection in some lineages. There were several instances of a decrease or loss of activity for this protein family – *Tropidechis* (decrease), *Vermicella* (almost complete loss) and *Echiopsis* (no detectable activity). The genus *Pseudechis* possessed the highest levels of activity for PLA_2_. We could find no evidence of correlation with any particular dietary categories to the activity for this protein family. Overall activity for this protein family was remarkably clade specific with only a small number of exceptions all in closely related species. These are discussed below:

Our results for the genus *Hoplocephalus* showed strong non-overlapping inter-specific variation in PLA_2_ activity for the three species, which co-varied with phylogenetic distance [43]. This was the only genus in our data set of 16 genera to show such a pattern. PLA_2_ activity has diverged substantially in this genus, with extremely low activity levels detected in *H. bungaroides* and its sister species *H. stephensi*, while activity levels are substantially higher in *H. bitorquatus.* This suggests that the major decrease in PLA_2_ activity occurred after the divergence of *H. bitorquatus* and *H .bungaroides/stephensi*, but before the divergence of *H. bungaroides.* It would appear from this that the loss of a single protein family may not incur a significant fitness cost.

**Fig. 7:**
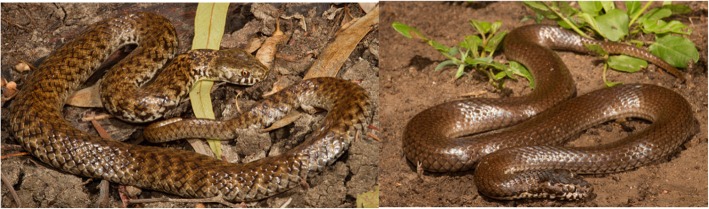
*Denisonia devisi* (left) and *D. maculata* (right). These two closely related frog specialists from the eastern sub-interior of Australia are indistinguishable based on scalation characters. Despite their similar diets, they possess highly divergent PLA_2_ activity. The process responsible for this divergence is currently unknown (Photos courtesy of Brendan Schembri)

Another notable result in our analysis of PLA_2_ activity was the difference in activity between the sister species *Denisonia devisi* and *D. maculata*. These allopatric species are indistinguishable in scalation characters [44], and extremely similar in morphology (Fig. 7) and ecology [30]. However, there is no molecular data available to estimate their genetic distance. PLA_2_ activity levels recorded in these two species showed an almost ten-fold difference with higher activity in *D. maculata* compared to *D. devisi* (Fig. 2.) These species show considerable dietary overlap (88% frogs for *D. devisi* and 95% frogs for *D. maculata* [30], so there is a possibility that the divergence in venom phenotypes between these two species may not have been driven by positive selection for diet.

These sister species with their near identical diets and foraging ecologies would make excellent candidates for testing the relative influence of genetic drift vs positive selection on venom. This could be done by quantifying the ratio of substitution rates at non-synonymous and synonymous sites ω (dN/dS ratios) known as likelihood ratio testing for positive selection. An ω ratio greater than one indicates positive selection [15]. The possibility of venom proteomes becoming radically “reset” onto new evolutionary trajectories due to genetic drift has received little attention in the literature and may have been underestimated.

We also found a marked divergence in PLA_2_ activity between the sister-species *Austrelaps ramsayi* and *A. superbus* with *A. ramsayi* having activity four times higher (Fig. 2).

The difference in activity between the sister clades *Acanthophis* and *Pseudechis* may be explainable by the different foraging strategies of these two genera. Although both genera are dietary generalists, *Acanthophis* is perhaps the most highly divergent Australian terrestrial elapid in its morphology and ecology, while *Pseudechis* is a generalized forager lacking morphological specializations. As *Acanthophis* is a slow-moving ambush predator, its lower PLA_2_ activity may be the result of selection pressure favouring a faster acting protein family e.g. post-synaptic neurotoxins (3FTxs), which have been shown to be the dominant protein family in the venom proteome of this genus [6]. This may make *Acanthophis* an example of selection pressure for venom activity not being driven by diet, but dictated by the requirements of foraging strategy.

### LAAO activity

LAAO activity showed greater clade specificity than activity levels of PLA_2_, suggesting that it is a more conservative protein family in Australian elapid venoms. LAAO showed early accelerated rates of change. This may indicate differential selection pressure as PLA_2_ is known to make up a much greater proportion of the venom proteome of nearly all snakes tested worldwide [3]. The early burst of evolution for LAAO accounts for the major differences in activity across the major elapid clades, but relatively smaller difference within them. In *Demansia* and *Pseudechis*, high activity has been retained in all species tested in these two genera. Activity is completely absent in the *Vermicella* and *Pseudonaja*/*Oxyuranus* clades. The loss of activity for LAAO occurred before the divergence of these two clades supporting the molecular evidence for relationships among these disparate taxa. The *Notechis* clade showed a trend for decreasing activity for LAAO in the genera *Austrelaps* and *Tropidechis*, and a unique instance of a recent shift in this protein family in the genus *Hoplocephalus.* High LAAO activity was recorded for all three species of *Hoplocephalus*, suggesting that the increase in activity occurred early in the evolutionary history of this genus, before the divergence of the three species. The absence of LAAO activity in some individuals in the genera *Parasuta* and *Suta*, suggests that the loss of this protein family may not incur a significant fitness cost.

Our dietary analysis does show a slight correlation for high activity for this protein family with a frog diet, but there were several important exceptions to this e.g. *Demansia psammophis* and *Hoplocephalus stephensi* (see dietary table Additional file section). In addition, this may also be a chance finding considering the number of snakes and the number of different diets included in the analysis.

### Limitations

Although this study represents the most comprehensive characterization of venom activity among Australian elapids to date, incorporating almost all major clades, it still did not sample all species of Australian elapids, or representatives of every major clade. Additionally we were unable to obtain molecular data to clarify the divergence time between two sister species in the genus *Denisonia* which possessed different activities for PLA_2_. Another limitation was not being able to compare other important toxin families such as metalloproteases, serine proteases, three-finger toxins and disintegrins. Further, we only screened LAAO activity against L-leucine and not its full substrate repertoire, so its activity against other amino acids is unknown and potentially could alter the results presented here.

As the majority of the snakes used in the study were already being held in captivity, this raises the issue of venom activity/composition being altered due to captive conditions or artificial diet. Based on previously published studies this should not significantly impact the results of this study [45–47]. For example, a study on the Jararaca *Bothrops jararaca* [48] found that venom is not significantly influenced by captivity. Another study on the same species [49], found that electrophoretic profiles, enzymatic activity and lethality showed only minor differences between captive and wild-caught specimens. A study on the Australian eastern brown snake *P. textilis* [50] also found that captivity had limited effects on venom composition.

We would like to emphasize that although we found no correlation between activity of these two protein families and broad dietary categories, this does not rule out the possibility of toxins within these protein families being adapted for prey specific toxicity. This is almost certainly the case as it has been established that PLA_2_ activity in taipans is not correlated to toxicity [51, 52]. Our study is just establishing a strong correlation between catalytic activity and phylogeny, the lack of correlation we observed between activity and diet is likely to prove less informative.

## Materials

### Snake venom

The study included 91 individual snakes plus pooled venom from two species of 39 species of Australasian elapids from the approximately 100 species of the terrestrial Australian elapids. This represents 17 of the 24 genera and eight of the 11 currently recognized clades. The localities and collectors for each snake are listed in Additional file 1: Table S2 (Supplementary Section).

All snakes were maintained at Venom Supplies Tanunda South Australia. Only adult snakes were used for the project. After milking, venom was freeze-dried and the lyophilized venom was reconstituted the day of the experiments in millipure water for LAAO assays and 4-nitro-3-octanoyloxybenzoic acid (NOB) buffer for PLA2 assays. All venom used for the study was from individual snakes except for *Oxyuranus temporalis* (pooled from two individuals from Ilkurlka Roadhouse W.A.), and *Oxyuranus microlepidotus* (pooled from several individuals from Goyder’s Lagoon S.A.). Most of the snakes used in the study were already in captive maintenance at Venom Supplies South Australia apart from two individuals of *Hoplocephalus bungaroides* in the private collection of Simon Tresseder Licence No. AKL 68528. The remaining 29 snakes were collected under the approval of animal ethics No A-2016-609 and scientific licences SL 101728 N.S.W. WISP 1747976 QLD and 08–000297 W.A. (Additional file 1: Table S1) (Snakes were transported by air to Venom Supplies, Tanunda South Australia.

### Reagents

The following materials were used for the study: NOB (4-nitro-3-octanoyloxybenzoic acid) Cat. No. BML-ST506–0050 Enzo Life Sciences, Leucine Pcode 1,001,836,926 L8000-25G Sigma Life Sciences, o-Dianisidine Pcode 1,001,844,919 D9143-5G Sigma Life Sciences, Horseradish peroxidase Pcode 1,002,325,511 P6782-5MG Sigma Life Sciences, acetonitrile HPLC grade Item# 20060.320 VWR chemicals, TRIS (Hydroxymethyl methylamine) 2311-500G Ajax Finechem, hydrogen peroxide UN No 2014 Biolab scientific, NaOH A482-500G Ajax Finechem.

For both assays reactions were monitored in a Synergy HT UV spectrophotometric plate reader, using Thermo Fischer Scientific 96 well clear-bottom microplates.

## Methods

### Phospholipase A2 activity

The protocol used followed Petrovic et al. 2001 [53]. PLA_2_ activity was measured using a kinetic assay that detects a chromogenic substrate NOB (4-nitro-3-octanoyloxy-benzoic acid). PLA_2_ enzymes cleave an ester bond resulting in the conversion of the NOB substrate to a fatty acid and the chromophore 4-nitro-3-hydrobenzoic acid. There is a linear relationship between the formation of the chromophore and absorbance which is monitored by a spectrophotometer.

Lyophilized venom was reconstituted in buffer at a concentration of 1 mg/mL or 100 μ/mL, depending on the potency of the PLA_2_ activity for each venom. Buffer solution was made up from 1 mL 1 M Tris buffer (pH 8.4), 2.5 mL 4 M NaCl, and 2.5 mL 0.4 M CaCl_2_, made up to 100 mL with millipure water. NOB substrate was reconstituted in acetonitrile at a concentration of 4 mg/mL. The buffer solution and the NOB substrate were mixed at a ratio 85:15 NOB buffer/NOB substrate. We used 100 μL of buffer solution as a negative control and 100 μL of *Pseudechis australis* venom as a positive control. *P. australis* was chosen as a positive control because its venom is known to contain large amounts of PLA_2_ toxins and preliminary assays showed it possessed high levels of PLA_2_ activity. Venom sample (100 μL) was added to five wells (to give *n* = 5) in a single column of a microplate, and in five wells in the adjoining column were added 100 μL of the NOB buffer/substrate mixture. This was preheated in a spectrophotometer at 37^o^ for 10 min. The venom was then added to the NOB buffer/substrate mixture and the reaction monitored every 30 s at 425 nm. The negative control absorbance measurements were subtracted from the absorbance measurements for the venom at the 10 min reading and the 0 min reading.

The equation obtained from the standard curve was then used to calculate the amount of chromophore generated and this value was divided by 10 to give nanomoles of product released per minute per mg of venom.

A standard curve of the amount of NOB product produced versus absorbance was created by alkaline hydrolysis using 4 M NaOH. Well A of a microplate was filled with 90 μL of water, 100 μL of 4 M NaOH and 10 μL of NOB substrate. The reaction was monitored in a spectrophotometer for 30 min until a stable plateau was apparent. The remaining wells in the column were then filled with 100 μL of diluent (mixture ratio 900 μL of water,1 mL NaOH and 100 μL acetonitrile). 100 μL of well A was then serially diluted 1:1 into the wells of the column. Absorbance was measured on a spectrophotometer. From the molecular weight of NOB substrate (309.3) and the amount applied to the well (conc. 4 mg/mL) we determined the amount per well in nanomoles. This gave the equation y = 0.0149X + 0.0524.

The standard curve was re-created *n* = 9 (*n* = 3 over 3 days), to check standard error at all points. Minimum resolution was determined to be 0.05 absorbance units (see Additional file section). Results were graphed using GraphPad Prism.

### L-amino acid oxidase activity

The protocol used followed Kishimoto and Takahashi 2001 [54]. The assay uses leucine as a substrate for the LAAO toxin in the venom which produces hydrogen peroxide and ammonia. A reagent mixture containing ortho-dianisidine and horse-radish peroxidase (HRP) is added. The hydrogen peroxide oxidizes the o-dianisidine to a coloured product, this is measured spectrophotometrically in a microplate reader at 450 nm.

Lyophilized venom was reconstituted in millipure water at a concentration of 100 μg/mL. The reagent mixture was made at the following ratio; leucine 600 μL, O-dianisidine 1.2 mL, HRP 120 μL and 25 mM Tris buffered saline pH 8.4 6 mL.

The venom samples (100 μg/mL) were added to five wells (to give *n* = 5), in a single column of a 96 well microplate at a volume of 50 μL per well. 200 μL of reagent mixture was added to five wells in an adjoining column. The plate was then pre-heated at 37 °C for 10 min in a spectrophotometer and the venom then added to the reagent mixture to start the reaction. The reaction was monitored once per min for 10 min. A negative control well contained 50 μL of Tris and a positive control well contained 50 μL of *Pseudechis australis* venom. This species was chosen as a positive control as preliminary assays showed it possessed high levels of LAAO activity. The negative control absorbance measurements were subtracted from the absorbance measurements for the venom at the 10 min reading and the 0 min reading.

The equation obtained from a standard curve was then used to calculate the amount of H_2_O_2_ generated and this value was divided by 10 to give nanomoles of H_2_O_2_ produced per minute per mg of venom (nmol/min/mg). Results were then graphed using GraphPad Prism.

A standard curve was created using H_2_O_2_ 9.79 M. This was diluted 1:10000 in millipure water to give a concentration of 0.979 mM. 50 μL of water were placed in wells B to H. To well A was added 50 μL of diluted H_2_O_2_. To well B was added 50 μL of diluted H_2_O_2_, which was then serially diluted 1:1 to wells B to G. All wells in the adjoining column contained 200 μL of the same reagent mixture used for the venom assays. The plate was pre-heated at 37 °C for 10 min and the two columns then mixed. Absorbance was monitored for 10 min at 450 nm. Amount of H_2_O_2_ in well A was calculated at 48.95 nmol. The amounts in the serially diluted wells were calculated from this and standard curve was created in GraphPad Prism giving the eq. 0.0276x + 0.01899. The standard curve was performed in triplicate.

### Comparative analyses of temporal dynamics and correlated evolution of PLA_2_ and LAAO profiles

For all analyses, mean PLA_2_ and LAAO activity levels for each species were used and logged to base 10 (after addition of 1 to avoid attempting to log 0 values); diet proportions were arcsine transformed [55]. To trace evolutionary changes in PLA2 and LAAO activity levels, these two variables were optimised on the most recent dated phylogeny of elapids (Lee et al. 2015), which included 28 of the species evaluated here. The relationships and divergence dates between these 28 species were derived by pruning out irrelevant taxa. Linear parsimony, square-change parsimony and maximum likelihood, as implemented in Mesquite [56], were used to reconstruct ancestral states at nodes and changes along branches. As all methods retrieved broadly similar patterns, the results and discussion focuses on the linear parsimony results.

To test whether rates of change in PLA2 and LAAO activity levels have been constant through time, or were faster/slower during the early phases of the elapid radiation, we implemented the delta transformation in BayesTraits, which either compresses or lengthens basal branches [57]. A delta of less than 1 indicates faster early rates of change, consistent with an early burst model. To evaluate whether changes in PLA2 and LAAO activity levels were correlated with shifts in diet, we obtained diet information for these 28 species from Shine [29–32, 35, 36, 38–41, 58–62], expressed as proportions of eight categories (invertebrates, fish, frogs, lizards, snakes, mammals, birds and eggs – see Additional file 1: Table S1 – Additional file Section). Phylogenetic comparative methods, implemented in BayesTraits continuous regression model, was used to test whether PLA2 or LAAO activity levels were correlated with any of these four diet items. A regression coefficient (beta) which is indistinguishable from 0 means no correlation.

All BayesTraits analyses used Markov-Chain Monte Carlo to infer the probability distribution of the target parameters, and analyses were repeated twice to confirm stationarity, with the results from both runs concatenated in Tracer [63] to generate mean and HPD statistics. The raw and transformed values for all these variables, and scripts for all analyses, are appended in Supplementary Information.

## Supplementary information

**Additional file 1: Table S1.** Diet of Australian elapids taken mainly from Shine. **Table S2**. Location sites and collectors for all snakes used in the study. **Figure S1**. PLA2 activity reconstructed using square-change parsimony, analytically equivalent to a special case of likelihood [42], on a phylogeny [1] of 16 of the 17 genera tested (27 of the 37 species; *Hemiaspis* has no PLA2 data). The Y-axis represents millions of years before present. Values had 1 added before log transformation (to avoid attempting to log 0 values, as any species with activity below the threshold of detection of 0.5 - unlogged value - was allocated a score of 0). Dotted vertical lines separate clades. Warmer colours (red) show higher activity; black is no activity; white is extreme uncertainty due to missing data in *Hemiaspis.* **Figure S2**. LAAO activity reconstructed using linear parsimony on a phylogeny [1] for all 17 genera tested (28 of the 39 species for which activity was measured). Y-axis represents millions of years before present. Values had 1 added before log transformation (to avoid attempting to log 0 values, as any species with activity below the threshold of detection of 0.5 - unlogged value - was allocated a score of 0). Dotted vertical lines separate clades. Warmer colours (red) show higher activity, black is no activity.

## Data Availability

The datasets used and/or analysed during the current study are available from the corresponding author on reasonable request.

## Abbreviations

HPD: highest posterior density
LAAO: L-amino acid oxidase
nmol/min/mg: nanomoles of chromophore produced per minute per milligram of venom
PLA_2_: Phospholipase A_2_
