# Supplementary material for: Activity of two key toxin groups in Australian elapid venoms show a strong correlation to phylogeny but not to diet
Source: BMC Evol Biol. 2020 Jan 13;20:9. doi: 10.1186/s12862-020-1578-x (PMC6958663; doi:10.1186/s12862-020-1578-x)
Supplement: Supplementary file 1 — Additional file 1: Table S1. Diet of Australian elapids taken mainly from Shine. Table S2. Location sites and collectors for all snakes used in the study. Figure S1. PLA2 activity reconstructed using square-change parsimony, analytically equivalent to a special case of likelihood [42], on a phylogeny [1] of 16 of the 17 genera tested (27 of the 37 species; Hemiaspis has no PLA2 data). The Y-axis represents millions of years before present. Values had 1 added before log transformation (to avoid attempting to log 0 values, as any species with activity below the threshold of detection of 0.5 - unlogged value - was allocated a score of 0). Dotted vertical lines separate clades. Warmer colours (red) show higher activity; black is no activity; white is extreme uncertainty due to missing data in Hemiaspis. Figure S2. LAAO activity reconstructed using linear parsimony on a phylogeny [1] for all 17 genera tested (28 of the 39 species for which activity was measured). Y-axis represents millions of years before present. Values had 1 added before log transformation (to avoid attempting to log 0 values, as any species with activity below the threshold of detection of 0.5 - unlogged value - was allocated a score of 0). Dotted vertical lines separate clades. Warmer colours (red) show higher activity, black is no activity. [file 12862_2020_1578_MOESM1_ESM.docx]

**Supplementary Section**

**Supplementary Table 1.** Diet of Australian elapids taken mainly from Shine.

|  | Invertebrates | Fish | Frogs | Lizards | Snakes | Mammals | Birds | Eggs | REF |
| --- | --- | --- | --- | --- | --- | --- | --- | --- | --- |
| Denisonia devisi | 0.025 | 0 | 0.88 | 0.095 | 0 | 0 | 0 | 0 | (30) |
| Denisonia maculata | 0.05 | 0 | 0.95 | 0 | 0 | 0 | 0 | 0 | (30) |
| Elapognathus coronatus | 0.03 | 0 | 0.53 | 0.44 | 0 | 0 | 0 | 0 | (55) |
| Parasuta flagellum | 0 | 0 | 0 | 1 | 0 | 0 | 0 | 0 | (29) |
| Parasuta monachus | 0 | 0 | 0 | 0.98 | 0.02 | 0 | 0 | 0 | (29) |
| Suta fasciata | 0 | 0 | 0 | 1 | 0 | 0 | 0 | 0 | (30) |
| Suta punctata | 0 | 0 | 0 | 0.83 | 0.17 | 0 | 0 | 0 | (30) |
| Suta suta | 0 | 0 | 0.03 | 0.67 | 0.02 | 0.19 | 0 | 0.09 | (29) |
| Cryptophis nigrescens | 0 | 0 | 0.01 | 0.915 | 0.055 | 0 | 0 | 0.02 | (31) |
| Cryptophis pallidiceps | 0 | 0 | 0.055 | 0.89 | 0.055 | 0 | 0 | 0 | (31) |
| Echiopsis curta | 0.015 | 0 | 0.32 | 0.52 | 0 | 0.13 | 0.015 | 0 | (39) |
| Austrelaps labialis | 0.04 | 0 | 0.17 | 0.754 | 0 | 0.018 | 0 | 0.018 | (51) |
| Austrelaps ramsayi | 0.016 | 0 | 0.17 | 0.78 | 0.034 | 0 | 0 | 0 | (51) |
| Austrelaps superbus | 0 | 0 | 0.503 | 0.464 | 0.013 | 0.02 | 0 | 0 | (51, 56) |
| Hoplocephalus bitorquatus | 0 | 0 | 0.77 | 0.153 | 0 | 0.077 | 0 | 0 | (49) |
| Hoplocephalus bungaroides | 0 | 0 | 0 | 1 | 0 | 0 | 0 | 0 | (49) |
| Hoplocephalus stephensi | 0 | 0 | 0.11 | 0.445 | 0 | 0.445 | 0 | 0 | (49) |
| Notechis scutatus | 0 | 0.04 | 0.48 | 0.01 | 0 | 0.4 | 0.11 | 0 | (41, 56) |
| Tropidechis carinatus | 0 | 0 | 0.4 | 0.0378 | 0 | 0.487 | 0.074 | 0 | (52) |
| Hemiaspis damelii | 0 | 0 | 1 | 0 | 0 | 0 | 0 | 0 | (35) |
| Demansia psammophis | 0 | 0 | 0.067 | 0.9 | 0 | 0 | 0 | 0.027 | (32) |
| Demansia torquata | 0 | 0 | 0 | 1 | 0 | 0 | 0 | 0 | (32) |
| Demansia vestigiata | 0 | 0 | 0.28 | 0.71 | 0 | 0 | 0 | 0 | (57) |
| Acanthophis antarcticus | 0 | 0 | 0 | 0.71 | 0 | 0.2 | 0.09 | 0 | (53) |
| Acanthophis pyrrhus | 0 | 0 | 0.083 | 0.75 | 0 | 0.166 | 0 | 0 | (53) |
| Pseudechis porphyriacus | 0 | 0 | 0.6 | 0.27 | 0.036 | 0.08 | 0 | 0 | (40, 41) |
| Pseudechis australis | 0.006 | 0 | 0.195 | 0.36 | 0.118 | 0.24 | 0.047 | 0.023 | (40) |
| Pseudechis butleri | 0 | 0 | 0 | 0.75 | 0.17 | 0.08 | 0 | 0 | (40) |
| Pseudechis papuanus | 0 | 0 | 0 | 0 | 0 | 1 | 0 | 0 | (40) |
| Pseudechis colletti | 0 | 0 | 0.25 | 0 | 0 | 0.75 | 0 | 0 | (40) |
| Pseudechis guttatus | 0.12 | 0 | 0.4 | 0.12 | 0.08 | 0.28 | 0 | 0 | (40) |
| Pseudonaja guttata | 0 | 0 | 0.41 | 0.35 | 0 | 0.24 | 0 | 0 | (50) |
| Pseudonaja modesta | 0 | 0 | 0 | 0.97 | 0 | 0.03 | 0 | 0 | (50) |
| Pseudonaja textilis | 0 | 0 | 0.08 | 0.51 | 0 | 0.41 | 0 | 0 | (50) |
| Vermicella annulata | 0 | 0 | 0 | 0 | 1 | 0 | 0 | 0 | (38) |
| Oxyuranus microlepidota | 0 | 0 | 0 | 0 | 0 | 1 | 0 | 0 | (36) |
| Oxyuranus scutellatus | 0 | 0 | 0 | 0 | 0 | 0.95 | 0.05 | 0 | (36) |
| Oxyuranus temporalis | 0 | 0 | 0 | 0 | 0 | 1 | 0 | 0 | (58) |

**Supplementary Table 2**. Location sites and collectors for all snakes used in the study.

| **Species** | **Specimen I.D.** | **Location** | **Collector/Keeper** |
| --- | --- | --- | --- |
| *Acanthophis antarcticus* | 78 | Smokey Bay S.A. | Nathan Dunstan |
|  | 241 | Smokey Bay S.A. | Nathan Dunstan |
|  | 80 | Middleback Range S.A. | Nathan Dunstan |
| *Acanthophis pyrrhus* |  | Alice Springs N.T. | Nathan Dunstan |
| *Austrelaps labialis* | 29 | Kangaroo Island S.A. | Nathan Dunstan |
|  | 30 | Kangaroo Island S.A. | Nathan Dunstan |
|  | 31 | Lobethal S.A. | Nathan Dunstan |
| *Austrelaps ramsayi* |  | Barrington Tops N.S.W. | Theo Tasoulis |
| *Austrelaps superbus* | 89 | Melbourne VIC | Nathan Dunstan |
|  | 90 | Melbourne VIC | Nathan Dunstan |
| *Demansia psammophis* |  | Newcastle N.S.W | Daniel Stace |
|  |  | Brisbane QLD | Richie Gilbert |
| *Demansia torquata* |  | Airlie Beach QLD | Kylee Gray |
| *Demansia vestigiata* |  | Bowen QLD | Nathan Dunstan |
|  |  | Rockhampton QLD | Callan Crigan |
| *Denisonia devisi* | DD5 | Glenmorgan QLD | Richie Gilbert |
|  | DD6 | Glenmorgan QLD | Richie Gilbert |
|  | DD7 | Glenmorgan QLD | Richie Gilbert |
|  | DD8 | Glenmorgan QLD | Richie Gilbert |
|  | DD9 | Glenmorgan QLD | Richie Gilbert |
| *Denisonia maculata* | DM1 | Eaglefield QLD | Brendan Schembri |
|  | DM2 | Eaglefield QLD | Brendan Schembri |
|  | DM3 | Eaglefield QLD | Brendan Schembri |
|  | DM4 | Eaglefield QLD | Brendan Schembri |
| *Echiopsis curta* | EC1 | s.e. of Perth W.A | Nathan Dunstan |
|  | EC2 | West coast S.A. | Nathan Dunstan |
|  | EC3 | West coast S.A. | Nathan Dunstan |
| *Elapognathus coronatus* |  | Jerramungup W.A. | Ross McGibbon |
| *Hemiaspis dameli* |  | Kogan QLD | Nathan Dunstan |
| *Hoplocephalus bitorquatus* | HB5 | 15km west of Dalby QLD | Nathan Dunstan |
|  | HB8 | 15km west of Dalby QLD | Nathan Dunstan |
|  | HB9 | Emerald QLD | Nathan Dunstan |
| *Hoplocephalus bungaroides* | 1 | Captive bred | Simon Tresseder |
|  | 2 | Captive bred | Simon Tresseder |
|  | BH3 | Captive bred | Captive bred |
| *Hoplocephalus stephensi* | W | Cooranbong N.S.W. | Stephan Mahony |
|  | B | Bunghwal N.S.W. | Theo Tasoulis |
| *Notechis scutatus* | 305 | Lake Alexandrina S.A. | Nathan Dunstan |
|  | 308 | Lake Alexandrina S.A. | Nathan Dunstan |
|  | 404 | Lake Alexandrina S.A. | Nathan Dunstan |
|  | 350 | Melbourne VIC | Nathan Dunstan |
|  | 353 | Melbourne VIC | Nathan Dunstan |
|  | 354 | Melbourne VIC | Nathan Dunstan |
|  | 444 | Heardsman Lake W.A. | Nathan Dunstan |
|  | 446 | Carnac Island W.A. | Nathan Dunstan |
| *Oxyuranus microlepidotus* | Pooled | Goyder’s Lagoon S.A. | Nathan Dunstan |
| *Oxyuranus scutellatus* | GL 258 | Gladstone QLD | Nathan Dunstan |
|  | C 419 | Cooktown QLD | Nathan Dunstan |
|  | SI 1 | Saibai Island QLD | Nathan Dunstan |
| *Oxyuranus temporalis* | Pooled | Ilkurlka Roadhouse W.A. | Nathan Dunstan |
| *Parasuta flagellum* |  | Cooma N.S.W. | Stephen Mahony |
| *Parasuta monachus* |  | Pilbara W.A. | Brian Bush |
| *Pseudechis australis* | 108 | Renmark S.A. | Nathan Dunstan |
|  | 128 | Renmark S.A. | Nathan Dunstan |
|  |  | Windorah QLD | Theo Tasoulis |
| *Pseudechis butleri* | PB3 | Cue W.A. | Nathan Dunstan |
|  | PB4 | Cue W.A. | Nathan Dunstan |
| *Psudechis colletti* | PC 45 | Captive bred | Nathan Dunstan |
|  | PC 46 | Captive bred | Nathan Dunstan |
| *Pseudechis guttatus* | 13 | Lake Broadwater QLD | Nathan Dunstan |
|  | 16 | Unknown | Nathan Dunstan |
| *Pseudechis papuanus* | PAP 1 | Saibai Island QLD | Nathan Dunstan |
|  | PAP 2 | Saibai Island QLD | Nathan Dunstan |
|  | PAP 3 | Saibai Island QLD | Nathan Dunstan |
| *Pseudechis porphyriacus* | 87 | Tanunda S.A. | Nathan Dunstan |
| *Pseudonaja guttata* | SB 20 | Ilfracombe QLD | Theo Tasoulis |
|  | SB 22 | Ilfracombe QLD | Theo Tasoulis |
| *Pseudonaja modesta* | PM 11 | Stonehenge QLD | Nathan Dunstan |
|  | PM 2 | Stonehenge QLD | Nathan Dunstan |
|  | PM 12 | 115km n of Meekatharra W.A. | Brian Bush |
|  | PM 13 | Carnarvon W.A. | Brian Bush |
|  | PM 14 | Carnarvon W.A. | Brian Bush |
| *Pseudonaja textilis* | S.A. 474 | Rowland Flat S.A. | Nathan Dunstan |
|  | A.S. 3 | Alice Springs N.T. | Nathan Dunstan |
|  | N.S.W. 1 | Singleton N.S.W. | Theo Tasoulis |
| *Rhinoplocephalus boschmai* |  | Charters Towers | Nathan Dunstan |
| *Rhinoplocephalus nigrescens* |  | Calliope QLD | Nathan Dunstan |
|  |  | Newcastle N.S.W | Theo Tasoulis |
| *Rhinoplocephalus pallidiceps* |  | Darwin N.T. | Nathan Dunstan |
| *Suta fasciata* | SF1 | 75km s of Karratha W.A. | Brian Bush |
|  | SF2 | Cue W.A. | Brian Bush |
|  | SF3 | Carnarvon W.A. | Brian Bush |
| *Suta punctata* | SP3 | 100km sse of Fitzroy Crossing W.A. | Brian Bush |
|  | SP4 | 150km s of Halls Creek W.A | Brian Bush |
| *Suta suta* | SS1 | QLD | Nathan Dunstan |
|  | SS2 | Mary Kathleen Dam QLD | Nathan Dunstan |
|  | SS3 | Dajarra QLD | Nathan Dunstan |
|  | SS4 | Mt Isa QLD | Nathan Dunstan |
|  | SS6 | Glenmorgan QLD | Nathan Dunstan |
| *Vermicella annulata* |  | Brisbane QLD | Richie Gilbert |

**Supplementary Fig 1**. PLA2 activity reconstructed using square-change parsimony, analytically equivalent to a special case of likelihood (42), on a phylogeny (1) of 16 of the 17 genera tested (27 of the 37 species; *Hemiaspis* has no PLA2 data). The Y-axis represents millions of years before present. Values had 1 added before log transformation (to avoid attempting to log 0 values, as any species with activity below the threshold of detection of 0.5 - unlogged value - was allocated a score of 0). Dotted vertical lines separate clades. Warmer colours (red) show higher activity; black is no activity; white is extreme uncertainty due to missing data in *Hemiaspis.*

**Supplementary Fig 2**. LAAO activity reconstructed using linear parsimony on a phylogeny (1) for all 17 genera tested (28 of the 39 species for which activity was measured). Y-axis represents millions of years before present. Values had 1 added before log transformation (to avoid attempting to log 0 values, as any species with activity below the threshold of detection of 0.5 - unlogged value - was allocated a score of 0). Dotted vertical lines separate clades. Warmer colours (red) show higher activity, black is no activity
